# Characteristics of the sputum microbiome in COPD exacerbations and correlations between clinical indices

**DOI:** 10.1186/s12967-022-03278-x

**Published:** 2022-02-05

**Authors:** Linfan Su, Yixian Qiao, Jinmei Luo, Rong Huang, Zhiwei Li, Hongbing Zhang, Hongmei Zhao, Jing Wang, Yi Xiao

**Affiliations:** 1 Department of Respiratory and Critical Care Medicine, Peking Union Medical College Hospital, Chinese Academy of Medical Sciences & Peking Union Medical College, Beijing, 100730 China; 2 Department of Pathophysiology, State Key Laboratory of Medical Molecular Biology Institute of Basic Medicine, Chinese Academy of Medical Sciences, School of Basic Medicine, Peking Union Medical College, Beijing, 100005 China

**Keywords:** Chronic obstructive pulmonary disease, 16S ribosomal RNA gene sequencing, Sputum microbiome

## Abstract

**Background:**

Chronic obstructive pulmonary disease (COPD) is a prevalent, progressive respiratory disease, and acute exacerbations of COPD (AECOPD) can accelerate the deterioration of the disease. Increasing evidence suggests that airway bacterial dysbiosis is associated with AECOPD. However, the exact relationship between changes in the sputum microbiome during AECOPD and clinical indices remains unclear.

**Methods:**

In this study, a total of 76 sputum samples were collected from patients with AECOPD (n = 28), stable COPD (n = 23), recovery (n = 15) and healthy controls (HCs; n = 10). The sputum microbiome profile was analysed by sequencing the V3‑V4 amplicon of the 16S rRNA (ribosomal RNA) gene.

**Results:**

The bacterial diversity (Shannon and Simpson’s index) was found to be significantly decreased in the AECOPD and recovery groups when compared to that in the stable COPD and HC groups. The most dominant phylum identified in the sputum samples of AECOPD patients was Proteobacteria, accounting for 30% of the microbiome. Compared to the stable COPD groups, the relative abundances of Firmicutes and Bacteroidetes were decreased, whereas those of Proteobacteria and Actinobacteria were increased in AECOPD patients. Furthermore, discriminative bacteria, such as *Haemophilus,* were identified as being specific taxa in AECOPD patients. Functional analysis showed that genes involved in membrane transport and signal transduction metabolism were enriched in the AECOPD group. Importantly, the proportions of *Veillonella* were positively correlated with lung function, and *Staphylococcus* was positively correlated with inflammatory indices.

**Conclusion:**

Our study revealed variations in the sputum microbiome of AECOPD (based on composition and function) in a Chinese cohort and highlighted its correlation to clinical indices. These results indicated that microbial dysbiosis may contribute to disease progression and provide microbial biomarkers for the diagnosis of AECOPD.

**Supplementary Information:**

The online version contains supplementary material available at 10.1186/s12967-022-03278-x.

## Background

Chronic obstructive pulmonary disease (COPD), one of the leading causes of morbidity and mortality worldwide, is characterized by irreversible airflow limitation due to airway and/or alveolar abnormalities [1]. Acute exacerbation of COPD (AECOPD) is defined as an acute worsening of respiratory symptoms, such as increased cough, purulent sputum production, and dyspnoea, which requires a change in treatment [2]. Frequent acute exacerbations lead to an increase in mortality as well as socioeconomic loss and health expenditures [3].

Bacteria-induced respiratory infection is the major cause of acute exacerbations of COPD [4]. Alterations in the airway microbiome are associated with decreased lung function and enhanced airway inflammation [5, 6]. Clinic indices such as spirometry indices are the indicators of the severity of airflow limitation and key to the diagnosis of COPD [7]. Inflammatory biomarkers can indicate bacterial exacerbation in COPD patients [8]. These clinical indices can be utilized to accurately profile disease severity and prognosis in COPD patients and may be influenced by microorganisms [9–11]. Recent evidence shows that different bacterial taxa identified in sputum samples from AECOPD patients are associated with different clinical outcomes [12]. However, the manner in which these detected bacteria are involved in pathogenesis and how they ultimately affect the clinical indices of the host remain unclear. Thus, it is necessary to investigate the correlation between the relating microbiota composition and the pathogenesis of acute exacerbation and clinical indices [13].

Traditional culture techniques have significant limitations regarding unculturable bacteria, as well as a poor sensitivity to detecting low-abundance bacteria [14]. In some situations, which mainly occurs for patients chronically colonized by *Pseudomonas aeruginosa*, chronic colonizing bacteria (rather than pathogenic bacteria) grow easily from the analysed sample by culturing, which can interfere with the potential cause of the exacerbations [15]. In recent years, advances in diagnostic technology, such as the 16S ribosomal RNA (rRNA) gene sequencing, have now allowed for the assessment of microbiome features with unprecedented personalization and precision, thus demonstrating alterations in the composition and relative abundance of bacteria related to COPD patients [16–18]. However, the exact relationship between changes in the sputum microbiome during AECOPD and clinical indices has not yet been elucidated by sequencing the 16S rRNA gene.

In this study, we investigated the correlation between key bacteria and certain clinical indices in COPD exacerbations by conducting 16S rRNA gene sequencing. We revealed that *Streptococcus* is the most dominant genus of AECOPD and found that *Veillonella* and *Staphylococcus* correlated with FEV1/FVC and C-reactive protein, respectively. These bacteria may serve as potential biomarkers and therapeutic targets for AECOPD.

## Methods

### Subject enrollment

From March 2019 to March 2021, 76 AECOPD patients who were admitted to the Respiratory Department of Peking Union Medical College Hospital and its affiliated hospitals were recruited for this study. Stable COPD patients and healthy controls were enrolled from outpatient clinics. Inclusion criteria were as follows: (a) diagnosed with COPD or AECOPD according to the GOLD criteria [1]; (b) age ≥ 40 years; (c) stable COPD patients were defined as clinically stable for at least 3 months free from acute exacerbation. Study exclusion criteria were: (a) a history of any other respiratory illnesses, such as asthma, pulmonary tuberculosis, concomitant bronchiectasis, sleep apnea syndrome, lung cancer, or interstitial lung disease; (b) underwent chronic treatment with oral corticosteroids or immunosuppressive drugs; (c) history of any antibiotics usage within the previous 4 weeks before enrollment. All AECOPD patients received antibiotics and inhaled corticosteroids (ICS) according to guidelines [19]. Patients in the recovery group were defined as 2 weeks post-therapy. They should meet discharge criteria and have discontinued antibiotics at the same time. All participants signed an informed written consent agreement.

### Sample collection and DNA extraction

Fresh sputum samples were collected from AECOPD patients at admission before any therapy and recovery patients discontinued antibiotics. We collected either spontaneous or induced sputa from each patient. Participants were asked to rinse their mouths with a 3% hypertonic saline solution before sampling. Induced sputum samples were then collected via inhalation of 3% hypertonic saline and expectoration of sputum into a sputum cup within 30 min [20]. A total of 76 specimens were assigned to four clinical states: 1. AECOPD (n = 28); 2. Stable (n = 23); 3. Recovery (n = 15); 4. Healthy controls (n = 10). Then the samples were divided into 0.5 ml aliquots and stored at − 80 ℃ for DNA extraction and the remaining part of the samples were submitted for routine bacterial culture.

Total genome DNA from all sputum samples was extracted using the CTAB method following the manufacturer’s instructions. DNA concentration and purity were monitored on 1% agarose gels. According to the concentration, DNA was diluted to 1 ng/µL using sterile water.

### Library preparation and sequencing

16S rRNA genes of distinct regions were amplified by a specific primer with the barcode. PCR reactions were performed using Phusion® High-Fidelity PCR Master Mix (NEB) as manufacturer’s instructions. Thermal cycling consisted of an initial denaturation (1 min at 98 ℃), followed by 30 cycles of denaturation (10 s at 98 ℃), annealing (30 s at 50 ℃), elongation (30 s at 72 ℃) and finally 72 ℃ for 5 min. Mix PCR products with the same volume of 1X loading buffer (SYB green) and operate electrophoresis on 2% agarose gel for detection. PCR products were then mixed in equidensity ratios and purified using Qiagen Gel Extraction Kit (Qiagen, Germany). Followed by the manufacturer's recommendations, sequencing libraries were generated by TruSeq® DNA PCR-Free Sample Preparation Kit (Illumina, USA), and then index codes were added. Library quality was assessed using the Qubit@ 2.0 Fluorometer (Thermo Scientific) and Agilent Bioanalyzer 2100 system. Finally, the library was then sequenced on the Illumina NovaSeq platform and subsequently generate 250 bp paired-end reads.

### Bioinformatics analysis

The 16S rRNA sequencing data of 76 samples were examined for paired-end reads assembly and quality control. Sequences analysis was performed by Uparse software (Uparse v7.0.1001) [21]. Sequences were assigned to the same OTUs with similarity ≥ 97%. A representative sequence from each OTU was selected for further annotation. To annotate taxonomic information, each representative sequence uses the Silva Database based on the Mother algorithm [22]. To study the difference of the dominant species in different samples and the phylogenetic relationship of different OTUs, multiple sequence alignment analysis was carried out by the MUSCLE software (Version 3.8.31) [23]. OTUs abundance data were normalized by a standard of sequence number consistent with the sample with the least sequences. Further analysis of alpha and beta diversity analyses were all calculated based on the output normalized data using QIIME software (Version 1.9.1). Alpha diversity analysis is applied in assessing the complexity of species diversity within a sample and included observed species, Chao1, Shannon, and Simpson indices. Beta diversity analysis was applied to explore the differences of bacterial communities between COPD patients at different stages and healthy controls in species complexity. PCoA analysis was carried out by stat packages, WGCNA package, and ggplot2 packages. Microbial metabolic function profiles were predicted using Phylogenetic Investigation of Communities by Reconstruction of Unobserved States (PICRUSt) software to generate the Kyoto Encyclopedia of Genes and Genomes (KEGG) pathways based on 16S rRNA gene sequence [24]. Finally, we calculated the Spearman correlation coefficient and corresponding *p*-value between each module and clinical traits.

### Statistical analyses

SPSS 21.0 software was used for basic statistical analysis of data, GraphPad Prism9 and R software (Version 2.15.3) were used for chart production (*p* < 0.05 was considered statistically significant). Normally distributed variables were presented as mean ± standard deviation and compared through one-way analysis of variance (ANOVA) followed by Tukey’s multiple comparison test. The nonparametric data were presented as median (interquartile range) and were analyzed by Kruskal–Wallis tests. Categorical variables were expressed as count and percentages (N, %) and compared using the χ^2^ test. Specifically, alpha diversity was calculated by the Wilcox rank-sum tests, and beta diversity was acquired by Adonis analysis. Use linear discriminate analysis effect size (LEfSe) to obtain different species between groups. In this study, the linear discriminant analysis (LDA) score threshold used was > 4.0. Spearman correlation analysis was performed to evaluate the relationship between the sputum microbiome and clinical indices and then use the pheatmap function in the pheatmap package for visualization.

## Results

### Characteristics of the study participants

A total of 76 sputum samples were collected from COPD patients at different stages of disease and healthy controls. The demographics and clinical features of the patients are summarized in Table 1. No statistical differences in age, gender, BMI, current smoker, comorbid conditions, medications, or exacerbation frequency were observed among the groups. The results of sputum culture showed no statistical difference may be due to the low positive rate. The smoking index was low in healthy people, but there was no significant difference among the COPD groups. As expected, lung function (FEV1%pred and FEV1/FVC values) was significantly decreased in all COPD patients when compared to that of healthy controls (*p* < 0.001). In addition, patients in the AECOPD group had a higher modified Medical Research Council (mMRC) dyspnoea score than stable COPD patients (*p* = 0.023). Blood routine parameters, glycemia, arterial blood gas analysis, inflammation index values (PCT and CRP), and GOLD classification were not found to be significantly different among the four groups.

**Table 1:** Major clinical characteristics of participants in this study

Characteristics	Healthy control (n = 10)	AECOPD (n = 28)	Recovery (n = 15)	Stable (n = 23)	*p* value
Age (years, mean ± SD)	66.3 ± 2.31	72.9 ± 10.19	73.4 ± 11.26	68.9 ± 7.92	0.121
Male (%)	5 (50.00)	19 (67.86)	11 (73.33)	20 (86.96)	0.155
BMI (kg/m^2^, IQR)	22.30 (21.71–24.69)	22.76 (21.36–24.58)	23.51 (20.76–25.95)	25.10 (22.49–28.33)	0.064
Smoking history (pack-years, IQR)	0.00 (0.00–5.88)	30.00 (4.38–40.00)	30.00 (15.00–46.00)	25.00 (1.50–30.00)	0.018*
Current smoker (%)	3 (30.00)	7 (25.00)	3 (20.00)	6 (26.09)	0.951
Comorbid conditions (%)					
Hypertension	3 (30.00)	12 (42.86)	10 (66.67)	10 (43.48)	0.286
Chronic kidney disease	0	2 (7.14)	1 (6.67)	0	0.493
Diabetes mellitus	1 (10.00)	4 (14.29)	3 (20.00)	2 (8.70)	0.769
Stroke	0	1 (3.57)	1 (6.67)	0	0.585
mMRC Dyspnoea scale (IQR)	NA	2.00 (1.00–3.00)	2.00 (1.00–4.00)	0.00 (0.00–2.00)	0.005*
FEV1% pred (mean ± SD)	96.97 ± 12.59	52.76 ± 21.58	50.23 ± 20.38	58.19 ± 21.42	< 0.001*
FEV1/FVC% (mean ± SD)	77.17 ± 3.43	53.26 ± 16.38	51.35 ± 13.91	50.80 ± 14.35	< 0.001*
White blood cell count (× 10^9^/L, IQR)	6.22 (5.43–7.06)	7.10 (5.29–8.64)	6.80 (5.10–8.75)	6.86 (5.64–8.41)	0.941
Blood Neutrophil count (× 10^9^/L, IQR)	3.54 (2.89–3.88)	4.59 (3.29–6.37)	5.57 (3.64–6.53)	4.74 (3.93–5.54)	0.271
Blood Eosinophil count (× 10^9^/L, IQR)	0.12 (0.05–0.41)	0.15 (0.04–0.26)	0.15 (0.08–0.20)	0.14 (0.06–0.20)	0.994
Hemoglobin (g/L, mean ± SD)	141.75 ± 26.59	136.86 ± 22.93	133.13 ± 24.89	151.41 ± 21.88	0.084
Platelet (× 10^9^/L, mean ± SD)	233.00 ± 56.06	220.18 ± 61.96	211.07 ± 80.02	222.09 ± 52.30	0.880
Glycemia (mmol/L, mean ± SD)	7.60 ± 2.00	7.67 ± 2.45	6.92 ± 2.88	5.98 ± 0.79	0.065
SCr (umol/L)	79.50 (57.25–129.00)	80.90 (60.00–90.00)	96.50 (58.00–103.00)	73.00 (65.5–80.25)	0.255
CRP (mg/L, IQR)	0.6 (0.32–7.39)	3.06 (1.52–7.22)	4.10 (1.00–12.38)	3.93(1.79–12.05)	0.549
PCT (pg/Ml, IQR)	NA	0.00 (0.00–0.00)	0.00 (0.00–0.00)	NA	0.926
PO_2_ (mmHg, IQR)	NA	79.30 (72.08–88.75)	87.00 (75.10–102.00)	80.00 (55.50–84.00)	0.159
PCO_2_ (mmHg, IQR)	NA	42.00 (36.78–48.38)	42.00 (36.50–52.50)	43.00 (39.00–57.00)	0.820
PH	NA	7.40 (7.37–7.42)	7.40 (7.38–7.43)	7.40 (7.36–7.42)	0.742
Sputum culture positive (%)	NA	8 (28.57)	4 (26.67)	1 (4.35)	0.071
Medications (%)					
ICS	NA	12 (42.86)	8 (53.33)	8 (34.78)	0.527
LABA/LAMA	NA	16 (57.14)	11 (73.33)	14 (60.87)	0.574
Frequency (%) (exacerbations in previous 1 year)					0.183
< 2	NA	15 (53.57)	7 (46.67)	17 (73.91)	
≥ 2	NA	13 (46.43)	8 (53.33)	6 (26.09)	
GOLD Classification (%)					0.943
I	NA	4 (14.29)	2 (13.33)	3 (13.04)	
II	NA	10 (35.71)	6 (40.00)	10 (43.49)	
III	NA	7 (25.00)	3 (20.00)	7 (30.43)	
IV	NA	7 (25.00)	4 (26.67)	3 (13.04)	

*BMI* body mass index, *mMRC* modified Medical Research Council, *FEV1* forced expiratory volume in 1 s, *FVC* forced vital capacity, *PCT* procalcitonin, *CRP* C-reactive protein, *SCr* serum creatinine, *ICS* inhaled corticosteroids, *LABA* long-acting beta-agonist, *LAMA* long-acting muscarinic antagonist, *GOLD* global initiative for obstructive lung disease, *NA* not applicable. *SD* standard deviation, *IQR* interquartile range

*p < 0.05

### 16S gene sequence‑based characterization

**Fig. 1:**
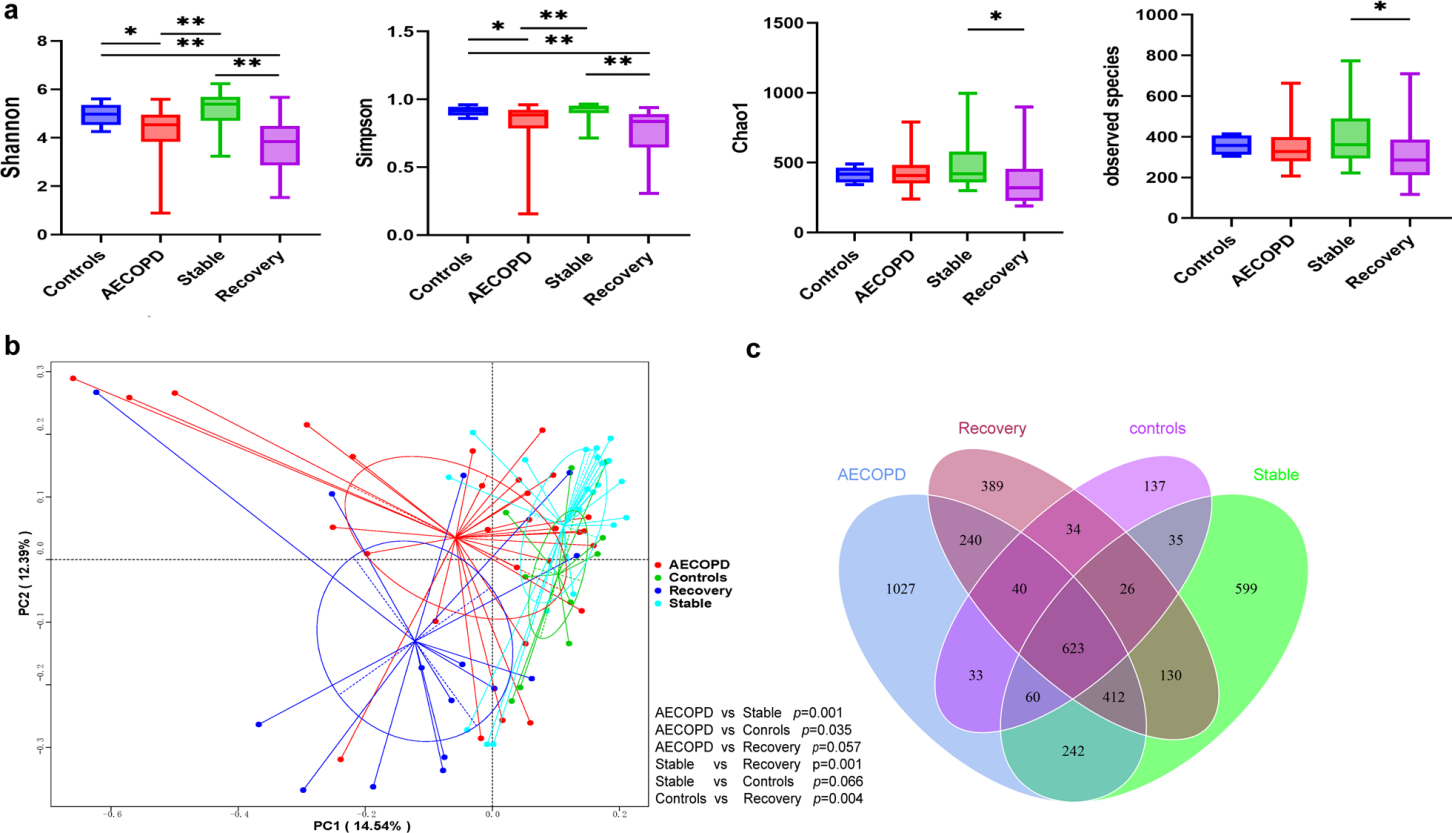
Bacterial community structure in healthy controls and COPD patients at different stages (AECOPD, recovery, and stable COPD, respectively). **a** Comparison of sputum microbiome alpha richness and diversity (Shannon index, Simpson’s index, Chao1, and Observed species) by Wilcoxon rank-sum test. *p < 0.05, **p < 0.01. **b** Principal coordinates analysis (PCoA) based on Bray–Curtis’s distance is represented. Each point represents one subject and different colors represent different groups. ADONIS analysis showed that the separation of bacterial communities was significant (*p* < 0.05) as described in the text, except for stable vs controls (*p* = 0.066), AECOPD vs recovery (*p* = 0.057). **c** Venn diagram showing different OTUs among groups

The samples yielded a total of 4053 operational taxonomic units (OTUs) at 97% sequence identity (2677, 1894, 2127, and 988 in the AECOPD, recovery, stable COPD, and healthy control groups, respectively). We measured alpha diversity to evaluate the diversity and richness of the bacterial community (Fig. 1a). The Shannon index and Simpson’s index represent microbial diversity, while the Chao1 index and observed species index reflect microbial richness. AECOPD samples had significantly decreased indices of diversity compared to those in the stable COPD and healthy control groups (*p* < 0.05). In addition, the AECOPD samples tended to have lower bacterial richness than the stable COPD and healthy control samples, but the differences were not statistically significant (*p* > 0.05). Samples collected from the recovery group had the lowest alpha diversity measures (*p* < 0.05).

In addition, we calculated the Bray–Curtis distance to investigate the clustering of microbial taxa in the four groups. Principal coordinates analysis (PCoA) showed that the separation of microbial communities was significant in the four groups (*p* < 0.05). Significant differences in microbial community structure were observed between the AECOPD and stable groups (*p* = 0.001), AECOPD and healthy controls (*p* = 0.035), the healthy control and recovery groups (*p* = 0.004), the stable COPD and recovery groups (*p* = 0.001) (Fig. 1b).

Venn diagrams distinguished between the sputum microbiomes of the four groups. As shown in Fig. 1c, there were 3467 OTUs in the AECOPD and stable COPD samples, of which 1337 (38.56%) were shared by the two groups; there were 2909 OTUs in the AECOPD and healthy controls samples, of which 756 (25.99%) were shared by the two groups; there were 3256 OTUs in the AECOPD and recovery samples, of which 1315 (40.39%) were shared by the two groups; there were 2830 OTUs in the recovery and stable COPD samples, of which 1191 (42.08%) were shared by the two groups; there were 2159 OTUs in the recovery and healthy controls samples, of which 723 (33.49%) were shared by the two groups; and there were 2371 OTUs in the stable COPD and healthy controls samples, of which 744 (31.38%) were shared by the two groups.

### Abundance of bacterial taxa changes among groups

**Fig. 2:**
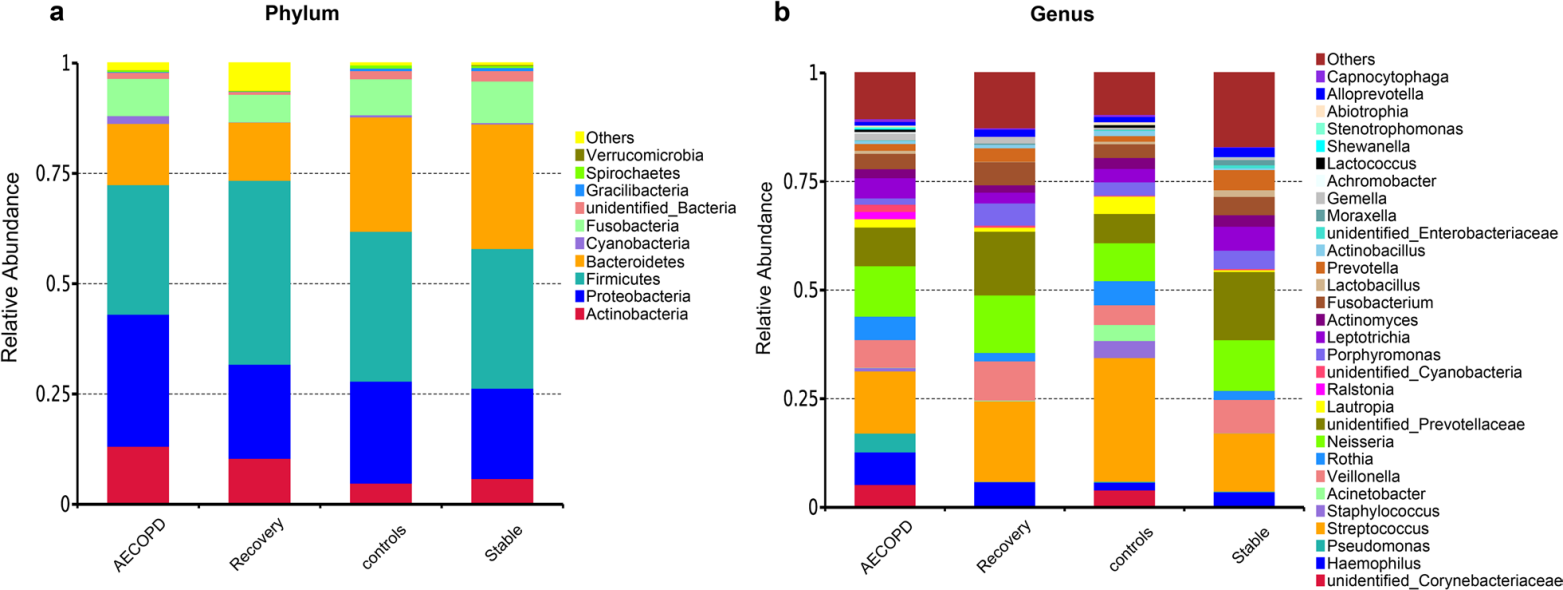
Relative abundance of the most prevalent bacterial phyla (**a**) and genera (**b**) in healthy controls and COPD patients at different stages (AECOPD, recovery, and stable COPD, respectively)

At the phylum level, the numbers of phyla detected in the AECOPD, recovery, stable COPD, and healthy control groups were 35, 29, 24, and 14, respectively. The dominant bacterial phylum in the AECOPD group was Proteobacteria (30.29%), followed by Firmicutes (29.85%) and Bacteroidetes (14.02%). In the stable COPD group, the major phylum was Firmicutes (31.63%), followed by Bacteroidetes (28.94%), and Proteobacteria (19.68%). In the recovery group, the major phyla were Firmicutes (44.04%), Proteobacteria (21.94%), and Bacteroidetes (13.35%). Lastly, in the healthy controls group, Firmicutes (34.01%), Bacteroidetes (26.01%), and Proteobacteria (23.09%) were predominant (Fig. 2a).

At the genus level, we detected 506, 399, 397, and 270 genera in the AECOPD, recovery, stable COPD, and healthy control groups, respectively (Fig. 2b). The dominant genera in the AECOPD group were *Streptococcus* (14.31%), *Neisseria* (11.60%), *unidentified_Prevotellaceae* (8.90%), *Haemophilus* (7.49%), and *Veillonella* (6.37%). In the stable COPD group, the dominant genera were *unidentified_Prevotellaceae* (15.69%), *Streptococcus* (14.31%), *Neisseria* (12.13%), *Veillonella* (7.38%), and *Haemophilus* (3.62%). In the recovery group, *Streptococcus* (27.32%) was the most highly represented genus, followed by *Neisseria* (8.69%), *unidentified_Prevotellaceae* (6.68%), *Veillonella* (4.35%), *unidentified_Corynebacteriaceae* (3.98%), and *Staphylococcus* (3.79%). *Streptococcus* was the most common genus in the healthy controls (18.41%), followed by *unidentified_Prevotellaceae* (14.63%), *Neisseria* (13.21%), *Veillonella* (9.00%), and *Haemophilus* (5.93%).

### Sputum microbiota alterations in AECOPD patients at the level of phylum and genus

**Fig. 3:**
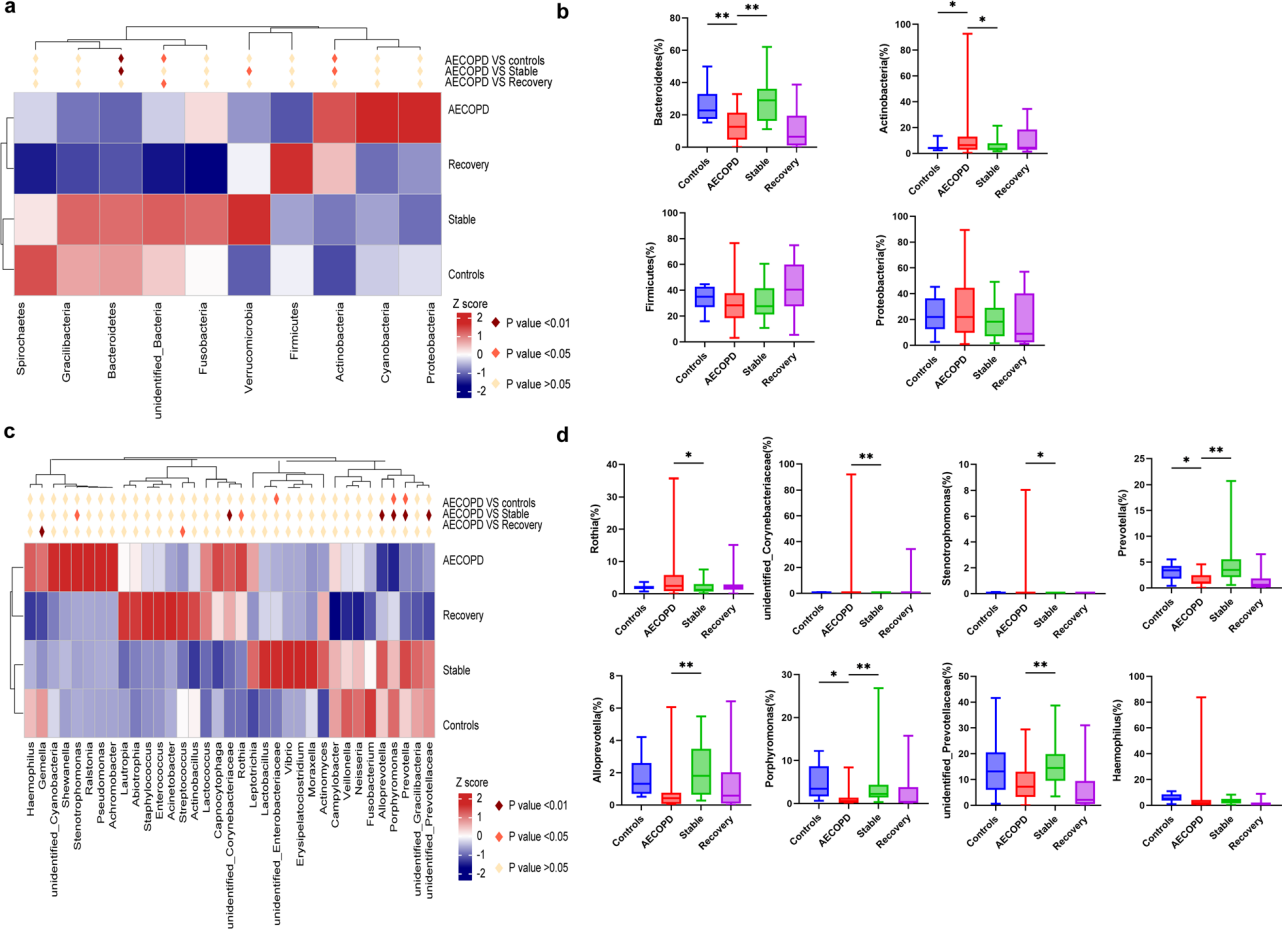
Heatmap and barplot showing the statistic differences of relative abundance of taxa among healthy controls, and COPD patients at different stages (AECOPD, recovery, and stable COPD, respectively) at phylum (**a**, **b**) and genus (**c**, **d**) levels. *, ** means *p* < 0.05, *p* < 0.01, respectively

At the phylum level, the relative abundance of Bacteroidetes was significantly decreased while those of Actinobacteria were increased in AECOPD patients compared to stable COPD and healthy controls (Fig. 3a, b). Although there were no statistical differences presented, we observed a decrease in Firmicutes and an increase in Proteobacteria in the AECOPD group compared to the stable COPD group. At the genus level, we observed significantly increased proportions of *Rothia*, *unidentified_Corynebacteriaceae*, *Stenotrophomonas,* and significantly decreased proportions of *Prevotella*, *Alloprevotella*, *Porphyromonas, unidentified_Prevotellaceae* in AECOPD patients compared to stable COPD(Fig. 3c, d).

### Bacterial taxonomic differences among the four groups

**Fig. 4:**
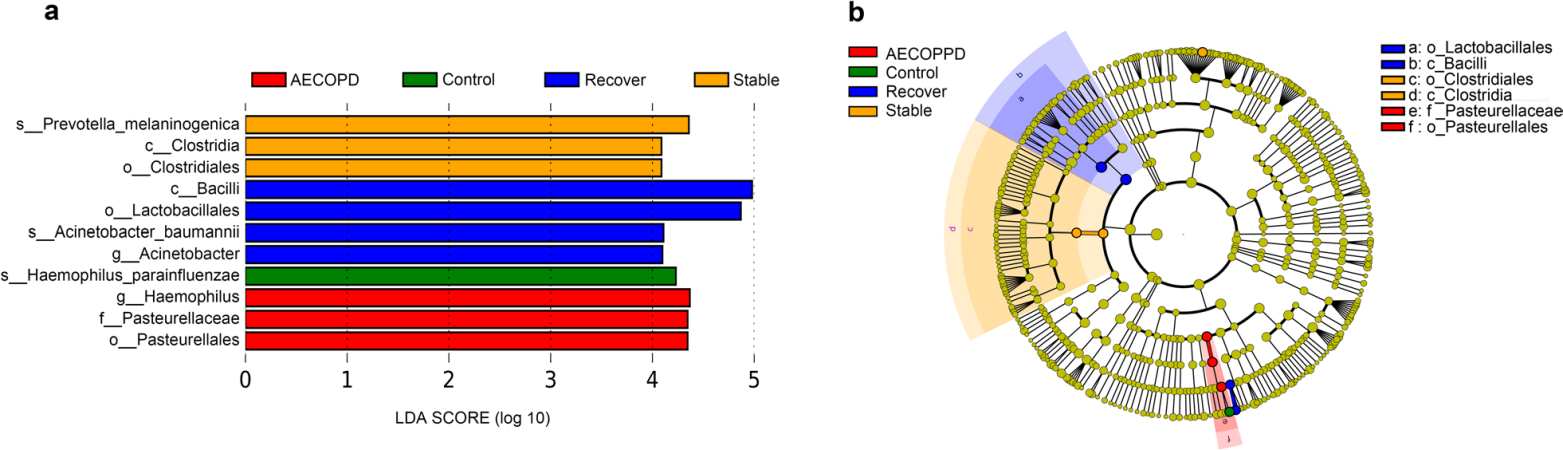
LEfSe analysis revealed the discriminative microbes in AECOPD, stable COPD, control, and recovery patient groups. **a** The histogram indicates the LDA score. These taxa showed statistically significant differences between the four groups (*p* < 0.05 by the Wilcoxon test). We selected the LDA threshold value of > 4 as significant in each group. **b** The cladogram of major differentially abundant taxa is based on LEfSe analysis. The circles radiating from inside to outside represent the taxonomic level from phylum to genus. Each small circle at a different classification level represents a classification at that level, and the diameter of the small circle is proportional to relative abundance. Different species biomarker follows the group for coloring. For example, the red node indicates the group of microorganisms that play an important role in the AECOPD group. The diameter of each circle represents the relative abundance of each taxon

To identify biomarkers from the disease groups, we performed a LEfSe analysis at the genera level. As expected, discriminative bacteria were identified in the four groups. We selected a linear discriminant analysis (LDA) to score higher than 4 to represent a significantly enriched genus in each group (Fig. 4a, b). The sputum microbiome of the AECOPD group was characterized by a dominance of *Haemophilus*, Pasteurellaceae, etc., whereas the microbiome in the stable COPD group was dominated by the genus *Prevotella*, Clostridiales, etc. (*p* < 0.05).

### Functional analysis of the microbiome in AECOPD patients by PICRUSt analysis

**Fig. 5:**
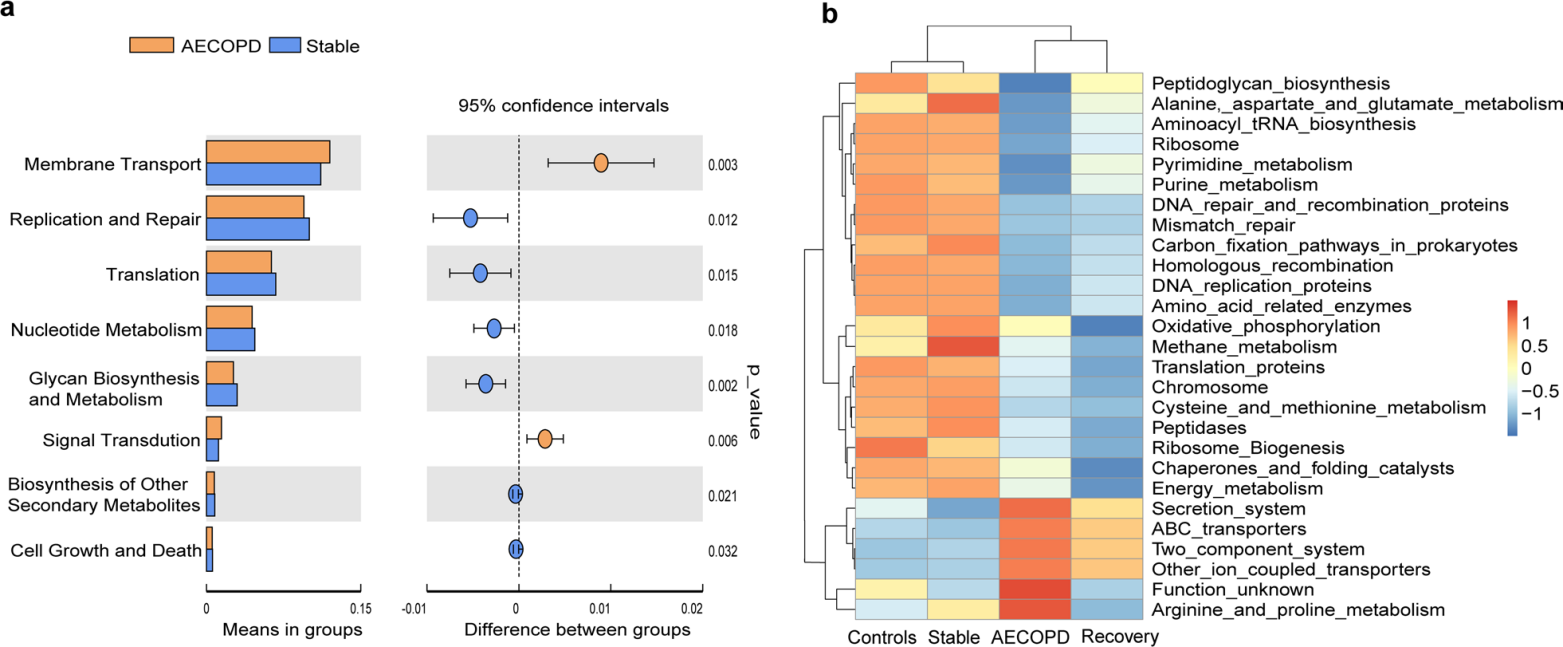
The differences in metabolic pathways by PICRUSt functional analysis. **a** Related KEGG pathways are plotted as a bar graph at KEGG level 2 between two groups. **b** Comparison of functional analysis in healthy controls and COPD patients at different stages at KEGG level 3

We performed a PICRUSt analysis based on the Kyoto Encyclopedia of Genes and Genomes (KEGG) database to evaluate sputum microbiome functions across groups in our study cohort [24]. Specifically, we found that the levels of metabolism, such as replication and repair, translation, nucleotide metabolism, glycan biosynthesis and metabolism, cell growth and death, and biosynthesis of other secondary metabolites, were decreased in the AECOPD group than in the stable COPD group. In contrast, metabolism related to membrane transport and signal transduction, including ABC transporters and secretion system were enriched in the AECOPD group (*p* < 0.05; Fig. 5a, b). The above results indicate that the sputum microbiome affects genetic information processing, nucleotide, and sugar metabolism.

### The sputum microbiome of AECOPD patients was associated with clinical indices

**Fig. 6:**
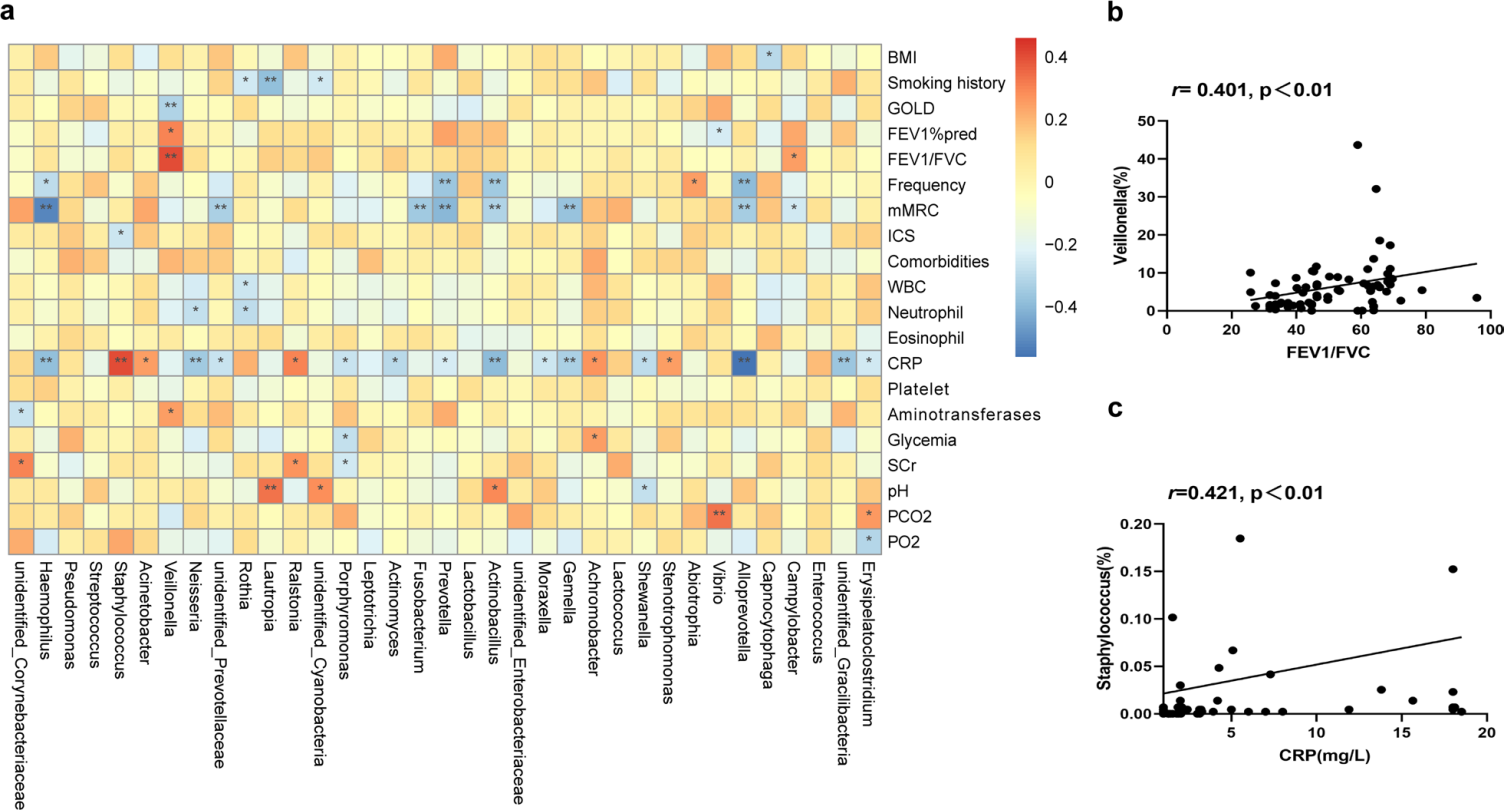
Spearman correlation between major sputum microbiome and clinical indices in COPD patients. **a** Heatmap of Spearman correlation analysis between the relative abundances of sputum microbiome and the clinical indices. *p < 0.05, **p < 0.01. **b** *Veillonella* shows a positive correlation with FEV1/FVC. **c** *Staphylococcus* shows a positive correlation with CRP

We used Spearman’s correlation analysis to evaluate the correlation between each group’s sputum microbiome and clinical index parameters, including the demographics and clinical features of the AECOPD patients, as shown in Fig. 6. Strong correlations (correction |r|> 0.4, *p* < 0.05) were found among 5 taxa and the 3 clinical indices in COPD subjects (Fig. 6a). Among the most abundant genera, the relative abundance of *Veillonella* exhibited a significant positive correlation with FEV1/FVC (Fig. 6b). *Staphylococcus* was positively correlated with inflammation index value CRP, whereas *Alloprevotella* was negatively correlated with CRP (Fig. 6c and Additional file 1: Fig. S1). Another finding was that *Haemophilus* and *Prevotella* were negatively correlated with the mMRC Dyspnoea scale (Additional file 1: Fig. S1).

## Discussion

In this study, we investigated the characteristics of sputum microbiota in patients with acute exacerbation and other disease states of COPD patients via cross-sectional observation by using high-throughput 16S rRNA sequencing. It was found that the lung microbiome of AECOPD and recovery patients demonstrated lower bacterial richness, as well as significantly different compositions, compared to those of the stable COPD and healthy control group subjects. We also found alterations in metabolic pathways, and common clinical indices were associated with AECOPD microbial communities. Collectively, these differences in our findings indicate that microbial dysbiosis may contribute to AECOPD and highlight its correlation to clinical indices, which could be considered microbial biomarkers used in potential targets for therapeutic interventions.

Our results demonstrated that microbiome diversity was reduced in AECOPD patients, compared with stable COPD patients and healthy controls. Samples collected from the recovery group had the lowest alpha diversity measures, which may likely be due to antibiotic exposure during clinical treatments. A large longitudinal study showed an overall reduction in microbial diversity during COPD exacerbations compared to samples from stable patients [25]. These results were consistent with the present study, thus suggesting that microbial diversity may be a biological indicator of AECOPD.

In our study, the most dominant phylum in sputum samples of AECOPD patients was Proteobacteria, which was consistent with the previous reports that the occurrence of AECOPD was related to the reduction in microbiome diversity and increased proportion of Proteobacteria [25, 26]. Proteobacteria and its members such as *Haemophilus* were associated with the onset of AECOPD [27]. In the present study, the most dominant phylum in sputum samples from the stable COPD group was Firmicutes, consistent with previous findings [28, 29].

At the genus level, the top 5 dominant genera present in all sputum samples were *Streptococcus*, *Neisseria*, *Prevotellaceae*, *Haemophilus,* and *Veillonella*, which has been reported in a previous study [28] except *Prevotellaceae*. In the present study, *Streptococcus* and *Prevotellaceae* were the most dominant genus in AECOPD and stable COPD groups respectively. *Streptococcus* has been reported as the most common genus among all samples in COPD patients [29, 30]. Currently, *Prevotellaceae* and *Streptococcus*, which both belong to the Firmicutes, are considered members of the core pulmonary microbiome [31].

Interestingly, compared to the stable COPD group, the AECOPD group had a higher level of Actinobacteria and the associated genera *Corynebacteriaceae* and *Rothia*. The AECOPD group also had a higher level of Proteobacteria and the associated genera *Stenotrophomonas* and *Haemophilus*, which are common COPD-related pathogens reported in the previous study [32, 33]. The level of Bacteroidetes and the associated genus *Prevotella* and *Porphyromonas* were markedly lower in the AECOPD group than in the stable COPD and healthy controls groups. It has been reported that the proportion of *Prevotella* of Bacteroidetes in the airways of asthma patients was lower than that of the normal population [34]. These results may indicate that alteration of normal flora distribution may lead to enhanced inflammation and increased exacerbation risk.

Functional prediction showed that the transporters, peptidases, purine/pyrimidine, and nucleotide metabolic pathways altered in the AECOPD group. For example, ABC transporters and the secretion system were enriched in AECOPD groups compared to the controls. ABC transporters and their role in substrate transport across the bacterial membrane have been associated with antibiotic resistance [35]. The bacterial secretion system contributes to secrete virulence factors for host invasion [36]. These pathways are important for the survival of pathogenic bacteria. Furthermore, AECOPD groups exhibited decreased glycan biosynthesis and metabolism relative to the stable COPD group and controls. Similar to previous findings, impaired glucose metabolism was also found in COPD patients [37]. The above results further indicate that the change in microbiome function may influence the occurrence or development of AECOPD.

In this study, our results indicated that the sputum microbiome was associated with disease indices. *Veillonella* was found to have a significant positive association with FEV1% (*p* < 0.05) and FEV1/FVC (*p* < 0.001). Additionally, *Staphylococcus* had a highly significant correlation with the inflammatory index CRP (*p* < 0.01). The proportion of *Veillonella* was slightly decreased in the AECOPD group (6.37%) when compared to that of the stable COPD group (7.38%) and healthy controls (9.00%) in our cohort. Simultaneously, the proportion of *Staphylococcus* was slightly increased in the AECOPD group (0.79%) compared to that of the stable COPD group (0.03%) and healthy controls (0.04%) in our cohort. Interestingly, Filho et al. found that a higher abundance of *Staphylococcus* was associated with decreased 1-year mortality while the survivors were abundant in *Veillonella* [12]. The results of our study may explain the reason for this result. *Veillonella* is a gram-negative anaerobic bacterium that belongs to normal oral commensals [38]. *Veillonella* species were observed to be greater in the healthy populations than in the COPD groups, thus suggesting a beneficial role for symbiosis in health and/or disease states [39]. *Staphylococcus*, a group of gram-positive pathogens that cause infections in the lungs [40], has been shown to result in the need for more antibiotics and longer hospitalizations [41]. *Staphylococcus aureus* in particular has been reported to directly trigger the formation of neutrophil extracellular traps (NETs), which may consequently influence the cycle of inflammation in COPD [42]. Taken together, these results indicate that *Veillonella* and *Staphylococcus* may be involved in COPD pathogenesis through their effects on lung function and local inflammatory responses, respectively. AECOPD patients may suffer a lung microecological imbalance, thereby aggravating the inflammatory responses and airflow limitations, which eventually increases the risk of readmission and mortality. Therefore, the sputum microbiome may be used to identify the clinical outcome and prognosis of COPD patients.

There were several limitations to our study. First, samples were cross-sectional and collected from different patients with different disease states, and the interference of confounding factors was unavoidable. Second, in addition to bacteria, viruses and fungi are also involved in the pathogenesis of acute exacerbations of COPD [33]. However, due to the lack of these data, we did not take them into account in the present study. Finally, our results were mainly restricted to the urban area of Beijing. Future large-scale multicentre studies with different biogeographical backgrounds are required to validate our findings.

## Conclusion

In conclusion, this study demonstrates that alterations in the abundance and composition of the sputum microbiome in COPD patients progress from stable to exacerbated states. Additionally, there are metabolic pathway alterations in COPD patients at different stages and the sputum microbiome is associated with common clinical indicators. These findings may provide new insight into the potential use of the sputum microbiome in AECOPD patients.

## Supplementary Information

**Additional file 1: Fig. S1.** Spearman correlations between major sputum microbiome with clinical indices in COPD patients. a. *Alloprevotella* shows negative correlation with CRP. *Prevotella* (b) and *Haemophilus* (c) show a negative correlation with the MRC Dyspnoea scale.

## Data Availability

The datasets used and/or analysed during the current study are available from the corresponding author on reasonable request.

## Abbreviations

COPD: Chronic obstructive pulmonary disease
AECOPD: Acute exacerbations of chronic obstructive pulmonary disease
HC: Healthy controls
BMI: Body mass index
FEV1: Forced expiratory volume in 1 s
FVC: Forced vital capacity
PCT: Procalcitonin
CRP: C-reactive protein
GOLD: Global initiative for obstructive lung disease
mMRC: Modified Medical Research Council Dyspnoea scale
WBC: White blood cell count
ICS: Inhaled corticosteroid
SCr: Serum creatinine
PCoA: Principal coordinates analysis
LEfSe: Linear discriminant analysis effect size
LDA: Linear discriminant analysis
