# Supplementary material for: Characteristics of the sputum microbiome in COPD exacerbations and correlations between clinical indices
Source: J Transl Med. 2022 Feb 5;20:76. doi: 10.1186/s12967-022-03278-x (PMC8818176; doi:10.1186/s12967-022-03278-x)

**Additional file 1: Figure S1**.

Spearman correlations between major sputum microbiome with clinical indices in COPD patients. **a.***Alloprevotella* shows negative correlation with CRP. *Prevotella* (**b**) and *Haemophilus* (**c**) show a negative correlation with the MRC Dyspnoea scale.


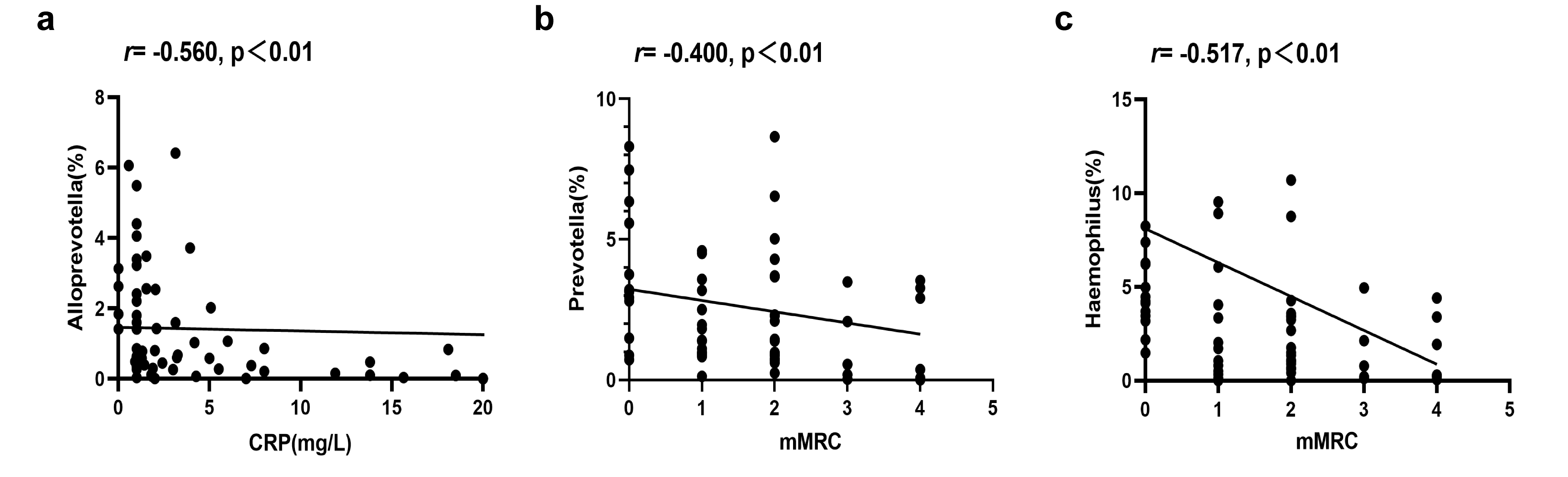

Supplement: Supplementary file 1 — Additional file 1: Fig. S1. Spearman correlations between major sputum microbiome with clinical indices in COPD patients. a. Alloprevotella shows negative correlation with CRP. Prevotella (b) and Haemophilus (c) show a negative correlation with the MRC Dyspnoea scale. [file 12967_2022_3278_MOESM1_ESM.docx]
